# Design and Control of a Series–Parallel Elastic Actuator for a Weight-Bearing Exoskeleton Robot

**DOI:** 10.3390/s22031055

**Published:** 2022-01-29

**Authors:** Tianshuo Wang, Tianjiao Zheng, Sikai Zhao, Dongbao Sui, Jie Zhao, Yanhe Zhu

**Affiliations:** State Key Laboratory of Robotics and Systems, Harbin Institute of Technology, Harbin 150001, China; 1110810113@hit.edu.cn (T.W.); zhengtj@hit.edu.cn (T.Z.); 16b908056@stu.hit.edu.cn (S.Z.); suidongbao@hit.edu.cn (D.S.); jzhao@hit.edu.cn (J.Z.)

**Keywords:** exoskeleton robot, series–parallel elastic actuator, cascaded PID control, sliding mode control, disturbance observer

## Abstract

Weight-bearing exoskeletons are robots that need to carry loads and interact with humans frequently. Therefore, the actuators of these exoskeletons are supposed to be capable of outputting sufficient force with high compliance and little weight. A series–parallel elastic actuator (SPEA) is designed, in this work, to meet the demanding requirements of an exoskeleton robot called PALExo. A gas spring is installed in parallel with an electric cylinder to adjust the force output range of the actuator according to the needs of the exoskeleton. A series elastic module (SEM) is installed in series with the electric cylinder and gas spring to improve the compliance of the actuator, the stiffness of which is variable to adapt to the different stiffness requirements of the exoskeleton’s legs in the standing phase and swinging phase. A force controller combining dynamic compensation and a cascade control with an inner velocity loop and a disturbance observer is designed for the SPEA. The performance of the force controller is verified by experiments and the results demonstrate that the controller has good adaptability to the stiffness of the SEM.

## 1. Introduction

An exoskeleton is a kind of wearable robot that is developed to help human body motion. The study of lower-limb exoskeletons was started by General Electric in the United States in the 1960s [1]. Since then, exoskeletons have been developing prosperously. Compared with conventional robots that perform repetitious tasks with great speed and precision, exoskeletons focus more on lightweight structures and interaction with humans. To this end, the actuators of exoskeletons must output sufficient force with high compliance and little weight.

Active compliance control, in which the compliance of stiff actuators is achieved by designing motion control systems, is a feasible method of achieving compliance. However, its performance and stability are greatly affected by the environment [2,3,4]. Series elastic actuators (SEAs), with their low impedance and high force fidelity, are attractive for exoskeletons. Unlike stiff actuators, SEAs contain an elastic element in series with a mechanical energy source [5]. Since the concept of SEAs was proposed in [6], SEAs have received much attention as next-generation actuators, especially in recent years [7]. Wyeth G et al. designed a revolute SEA with four linear springs intended for use in human–robot interaction applications, and this structure is still used in the design of some compliant actuators in recent years [8]. The motor of this SEA was treated as a velocity source for the elastic element, rather than as a torque source [9]. J. F. Veneman et al. designed a series elastic- and Bowden cable-based actuation system and applied it to the gait rehabilitation robot LOPES, of the University of Twente [10]. In [11], a revolute SEA with a torsional spring was designed and applied to an active orthosis system. An inner-loop position control, based on a proportional–derivative (PD) controller, feed-forward control, and a disturbance observer (DOB) were employed to robustly control this SEA in environments interacting with a human. In [5], a prismatic SEA with compact size was designed, in which the spring was placed between the motor housing and the chassis ground.

Traditionally, SEAs use linear springs as their elastic elements because they are cheap and follow Hooke’s law for force estimates. However, the use of linear springs introduces a trade-off in the selection of spring stiffness. Soft springs produce high-fidelity force control and low output impedance, but limit force range. Stiff springs provide high force range but reduce force fidelity [7,12]. Variable stiffness actuators (VSAs) attempt to overcome this trade-off by tuning the stiffness of a passive mechanical element with a secondary motor [13,14]. However, these systems are often complicated and bulky, hampering their application in lightweight, wearable robots. Murat R et al. has presented a novel compact elastic actuator, in which the stiffness of the actuator is adjusted by varying the clutch length of the cylindrical beams in an off-line fashion. However, the stiffness of this actuator is constant once it is in operation [15]. Passive nonlinear springs (NLSs) encode single, nonlinear torque profiles, according to task demands, without complicated mechanical structures. N. Schmit et al. proposes a cable mechanism based on a non-circular spool that synthesizes a nonlinear rotational spring from a linear spring [16]. Austin J. et al. have designed a compact NLS by combining a variable-radius cam with a rubber elastic element and proposed a state observer that captures the hysteretic effects exhibited by the rubber to provide an accurate estimate of actuator torque [17]. The application of variable-radius cams complicates the mechanical structures of their NLSs. Additionally, it is difficult for a cam to realize a wide stiffness range. Hu B. et al. design a nonlinear series elastic cable driver; a triangular pulley block and a linear spring are used to form the nonlinear spring mechanism, simulating the passive elastic properties of human skeletal muscle [18]. Sariyildiz E et al. design a variable stiffness SEA by using soft and stiff springs in series, so as to relax the fundamental performance limitations of conventional SEAs [19]. In this design, a stiff torsional spring is placed between the motor and the transmission element and a soft linear spring is placed between the transmission element and the load. The different locations of soft and stiff springs made the dynamic model and control of the SEA complicated. Furthermore, the series arrangement of springs make the structure insufficiently compact. In this paper, a variable-stiffness series elastic element with three springs in series and parallel arrangements is designed according to the working conditions of weight-bearing exoskeletons.

Installing an elastic element in parallel with the actuators is an attractive idea, enabling the actuators to meet the output requirements of the exoskeleton with less weight. Parallel elastic actuators (PEAs), popularly used in legged robots [20,21] and exoskeletons [22,23] in the past decade, can significantly modify the range of the original actuators’ force or torque and have the potential to reduce the energy consumption of the original actuators. To reduce the energy consumption, the stiffness of the parallel elastic elements should be designed according to its known motion [24,25,26], or a switch—which can be a brake, a clutch, or a trigger mechanism—should be introduced to engage the spring when energy storage is desired and disengage it to avoid the spring’s force interfering with the desired joint motions [27,28,29]. The motions of weight-bearing exoskeletons are variable and their actuators are expected to be lightweight and simple, so it is difficult to reduce the energy consumption of weight-bearing exoskeletons by designing PEAs. In this paper, a gas spring is installed in parallel with the motor to modify the range of the motor’s force, regardless of energy consumption.

In recent years, some researchers planned to replace the mechanical springs in SEA and PEA with other technologies. Allen DP et al. designed a VSA that uses dielectric elastomer transducers for springs. It did not need mechanical stiffness-adjusting components; however, some of the weaknesses of dielectric elastomer transducer technology have not been addressed [30]. Mrak B et al. designed a magnetic spring as a fatigue-free alternative to mechanical springs in compliant actuators [31]. These technologies have certain advantages over traditional commercial springs in some respects, but their reliability and cost need to be improved. Therefore, commercial springs are chosen as elastic elements of the SEA and PEA in this paper.

The combination of SEAs and PEAs, having been used in several studies [32,33], increases the compliance of actuators and reduces actuator requirements. A powered ankle–foot prosthesis was designed by introducing a series spring and a unidirectional parallel spring to mimic the human ankle during level-ground walking. A series of series-parallel elastic actuators (SPEAs) was proposed to reduce motor torque and increase efficiency, in which several intermittent mechanisms were used to engage several parallel springs in sequence with the rotation of the motor [34,35,36]. This series of novel actuators are able to improve the torque performance of the motor. However, the complicated and bulky mechanical structures hamper their application. The investigations of SPEAs are fewer than those of SEAs and PEAs; especially, few applications of SPEAs are reported. In this paper, a SPEA is introduced in a weight-bearing exoskeleton.

The rest of the paper is organized as follows. Our previous work and the main design requirement of the SPEA is introduced in Section 2. After the design of the SPEA is described in Section 3, a control strategy based on sliding mode control (SMC) and disturbance observer (DOB) and a cascaded, proportional–integral–derivative (PID) control strategy with an inner velocity loop and DOB are proposed in Section 4. The performance of the control strategies is verified by several experiments in Section 5. Finally, Section 6 concludes the paper.

## 2. Background

**Figure 1:**
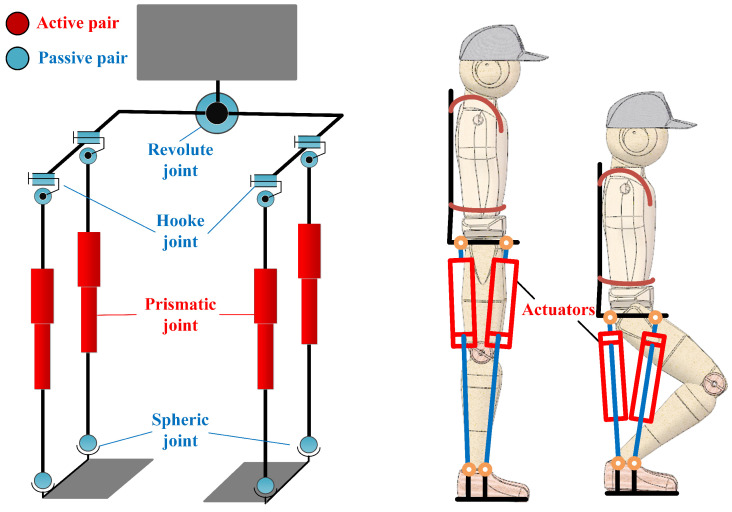
DOFs arrangement of PALExo.

An under-actuated parallel exoskeleton prototype, PALExo, has been developed in our previous work [37], the degrees-of-freedom (DOFs) arrangement is shown in Figure 1. Each leg of PALExo has two chains and each chain has three joints, including one passive universal joint, one active prismatic joint, and one passive spherical joint. In [37], the actuator of the exoskeleton is briefly introduced, and a force control method based on sliding mode control is proposed for the SEA without considering the parallel elastic element and different stiffness of the series elastic module. In this paper, the detailed design process and further research on force control of the SPEA will be introduced.

Exoskeletons, attached to human limbs when working, are a specific type of robot meant for interaction with human limbs. Therefore, exoskeletons are supposed to be lightweight and compliant. Moreover, weight-bearing exoskeletons, meant for carrying a heavy load for humans, are expected to have significant power, extending human strength. The actuators, which are the most important elements of PALExo, greatly affect the performance of PALExo. To make PALExo meet the above requirements, the actuators are supposed to be capable of a large range of telescopic movement and of outputting enough force with good compliance.

We expect the theoretical maximum carrying weight of PALExo is more than 80 kg and its weight is less than 30 kg. Additionally, to ensure flexibility, the maximum speed the PALExo’s leg motion is greater than 0.5 m/s. Under certain extreme working conditions, the center of mass (COM) of PALExo is directly above a chain of the parallel structure, and the total weight of the exoskeleton and load acts on a single actuator. Therefore, the actuators of PALExo should be able to output a force of 1078N and a telescopic motion with a speed of 0.5 m/s. Considering the performance and weight, we choose the telescopic cylinder (PC25: Thomson Inc., Radford, VA, USA) and the motor (APM-SB04A: Mecapion Inc., Daegu, Korea) as the actuator. Their main parameters are shown in Table 1.

The maximum motion speed and output force can be calculated as follows:

(1) $$V_{\max}=60\;r/s\times 0.01\;m/r=0.6\;m/s$$

(2) $$F_{\max}=2\times \pi \times 1.27\;N\cdot m÷0.01\;m/r=798\;N$$

Obviously, the actuator, being only composed of the telescopic cylinder and the motor, is not able to meet the output force requirements of PALExo. The actuators of the exoskeleton need to resist the impact when the foot is landing on the ground, and they interact with human body at all times. Therefore, the compliance requirements for PALExo of the actuators are very high. The actuator is only composed of stiff elements; compliance is achieved by a motion control system that cannot meet the compliance requirements of PALExo. Modifying the actuator by elastic elements is a feasible way to overcome this problem.

## 3. Design of the SPEA

**Figure 2:**
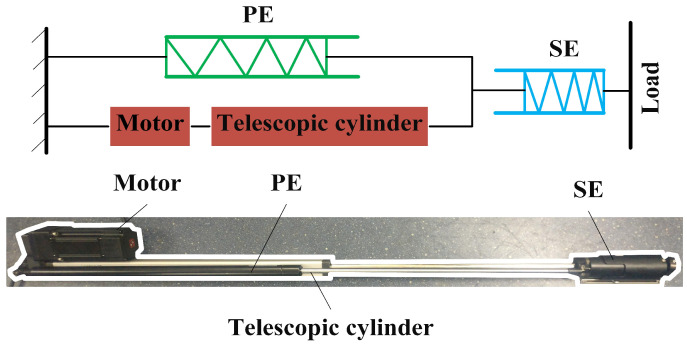
Series-parallel elastic actuator of PALExo.

It is well known that the skeletal muscles of humans are excellent actuators, which have good performance and compliance with small weight. There are many elastic elements in skeletal muscles playing a major role during motion. Hill proposed a macroscopic three-element elastic muscle model [38]. According to this model, a skeletal muscle is composed of a contractile element (CE), a series elastic element (SE) and a parallel elastic element (PE). The similarity between these three elements and the current state-of-the-art of compliant actuation concepts is clear. The skeletal muscles can be regarded as SPEAs. With reference to the muscle model, the stiff linear actuator mentioned above can be modified with series of elastic elements and parallel elastic elements, as shown in Figure 2.

### 3.1. Parallel Elastic Element

**Figure 3:**
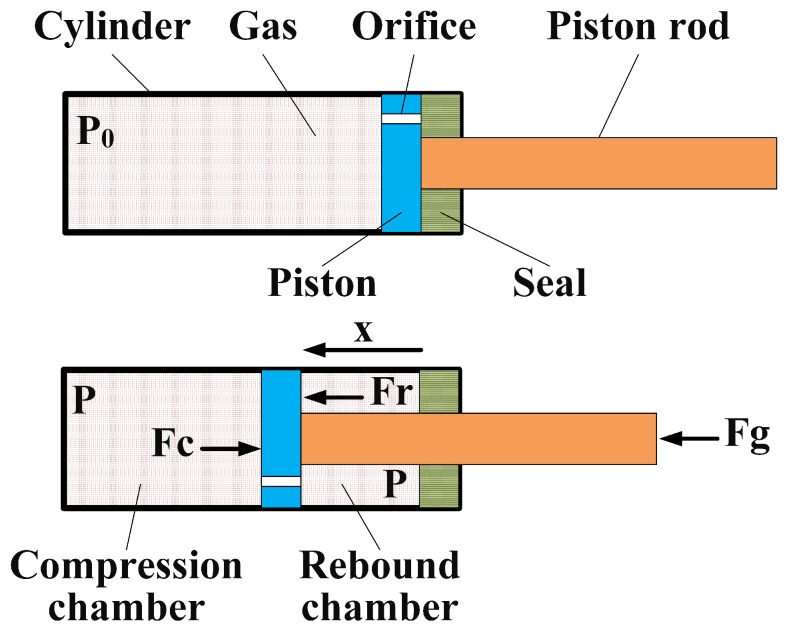
Schematics of gas spring.

Gas springs are elastic elements that output a force by means of compressed gas. They have been widely used in industry, especially for supporting weight, due to their characteristics. Gas springs have compact design and an excellent assembling ability, easily mounted with other applicatory products. One of their most important characteristics is to maintain the initial force, practically constantly, with only small variations, even for long strokes. A gas spring is composed of a cylinder with compressed gas, a piston, a piston rod and a seal, as shown in Figure 3. There are orifices on the piston, which causes the filling pressure to act on both sides of the piston. Gas springs produce an extension force because of area difference between two sides of the piston. The output force of a gas spring depends on the filling pressure and the area difference. The area difference is constant. However, the filling pressure is relative to the amount of compression (AOC) of the gas spring, because the movement of the piston rod changes the volume of the filling gas. The extension force of the gas spring can be calculated as shown in Equation (3).

(3) $$F_{g}=P\cdot \left(S_{pc}-S_{pr}\right)=P\cdot S_{rod},$$

where, $F_{g}$ represents the output force of the gas spring, *P* represents the pressure in the cylinder, $S_{pc}$ and $S_{pr}$ represent the area on the side of piston in the compression chamber and in the rebound chamber, respectively, and $S_{rod}$ represents the cross-sectional area of the piston rod.

Assuming that the temperature of the filling gas in the gas spring is constant, the pressure can be obtain as follows:

(4) $$P=P_{0}\cdot V_{0}/V=P_{0}\cdot L\cdot S_{pc}/\left(L\cdot S_{pc}-x\cdot S_{rod}\right),$$

where, *x* is the AOC of the gas spring, $P_{0}$ and $V_{0}$ respectively represent the pressure and volume of the cylinder when x=0, *V* is the volume of the cylinder, and *L* is the stroke of the gas spring. Combining Equations (3) and (4), we get:

(5) $$F_{g}=P_{0}\cdot L\cdot S_{pc}\cdot S_{rod}/\left(L\cdot S_{pc}-x\cdot S_{rod}\right)=F_{0}\cdot L\cdot S_{pc}/\left(L\cdot S_{pc}-x\cdot S_{rod}\right),$$

where, $F_{0}$ represents the initial output force of the gas spring when x=0.

A commercial gas spring, whose main parameters are shown in Table 2, is selected as the elastic element in parallel with the telescopic cylinder.

**Figure 4:**
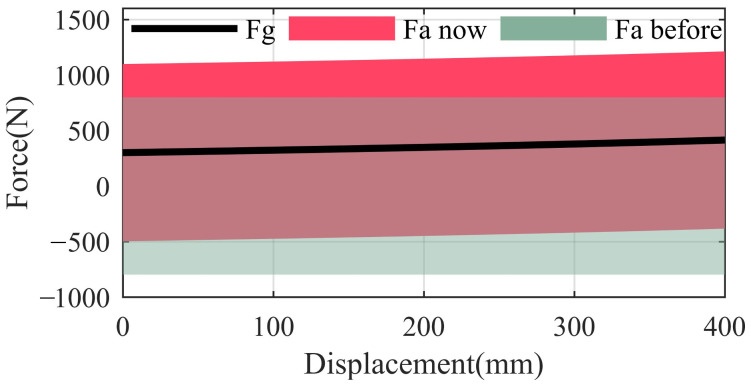
Effect of installing a gas spring in parallel with the actuator. Fg represents the output force of the gas spring; Fa now represents the force range of the PEA; and Fa before represents the original force range of the actuator. The extension force of the actuator is selected as positive force in this figure.

The gas spring, motor, and telescopic cylinder form a PEA. The output force of the gas spring and the force range of the PEA are obtained according to Equation (5); the result is shown in Figure 4.

In Figure 4, the force of the gas spring changes little (320 N 441 N) even if the AOC changes from 0 to 400 mm, which is difficult to realize by means of conventional metal springs. The symmetrical force range of the actuator is greatly modified by means of the gas spring. The force range changes from (−798 N 798 N) to (−478 N 1118 N) when the AOC is 0.

### 3.2. Series Elastic Element

**Figure 5:**
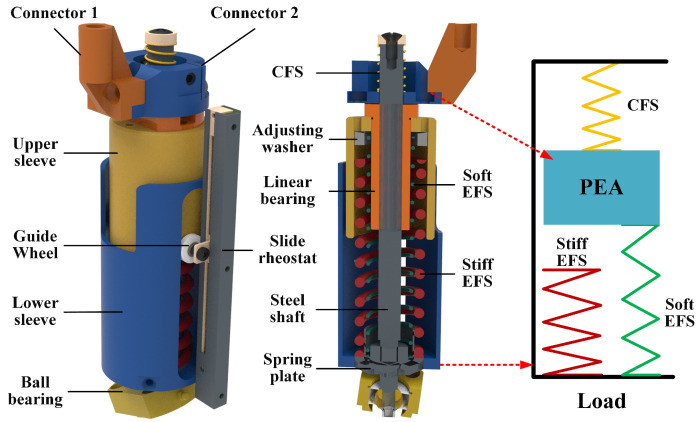
Mechanical structure of the SEM. The connector 1 and connector 2 in the figure is used to connect to the gas spring and the telescopic cylinder.

To achieve good compliance, an elastic element is introduced in series with the above-mentioned PEA. The stiffness of the series elastic element has significant effects on the performance of the actuator. Soft springs produce high fidelity of force control and low output impedance, but limit the force range. Stiff springs provide high force range but reduce force fidelity. The movement of the swing leg needs a high degree of flexibility. To achieve low output impedance, soft springs are preferred for the series elastic element of the swing leg. The stance leg needs to provide a large force when walking. Accordingly, stiff springs are preferred for the series elastic element of the stance leg. Obviously, it is difficult for a conventional series spring with constant stiffness to meet both the requirements of the swing leg and the stance leg of PALExo. To address this problem, a variable stiffness series elastic module (SEM) is designed, as shown in Figure 5, which is able to achieve large-span stiffness changes at certain positions.

The elastic elements of the SEM is composed of three linear metal springs. Two springs work mainly when the SEM is outputting an extension force, which are called “extension-force springs” (EFSs) in this work. Conversely, the other spring works mainly when the SEM is outputting a contraction force, and is called a “contraction-force spring” (CFS). A stiff EFS and a soft EFS are installed in parallel, and the stiff EFS is engaged only when the SEM has a particular AOC. The stiff EFS is mainly engaged in stance phase of PALExo’s legs. Conversely, the soft EFS and CFS are mainly engaged in the swing phase. There are two sleeves in the SEM applied to fix the EFSs, which are capable of axial relative motion by means of a linear bearing. Two small guide wheels on the upper sleeve and two chutes in the lower sleeve are used to limit rotation between the two sleeves around the axis. Several adjusting washers in the upper sleeve are used to adjust the actions of the stiff EFS. The AOC of the SEM is measured by means of a slide rheostat.

**Figure 6:**
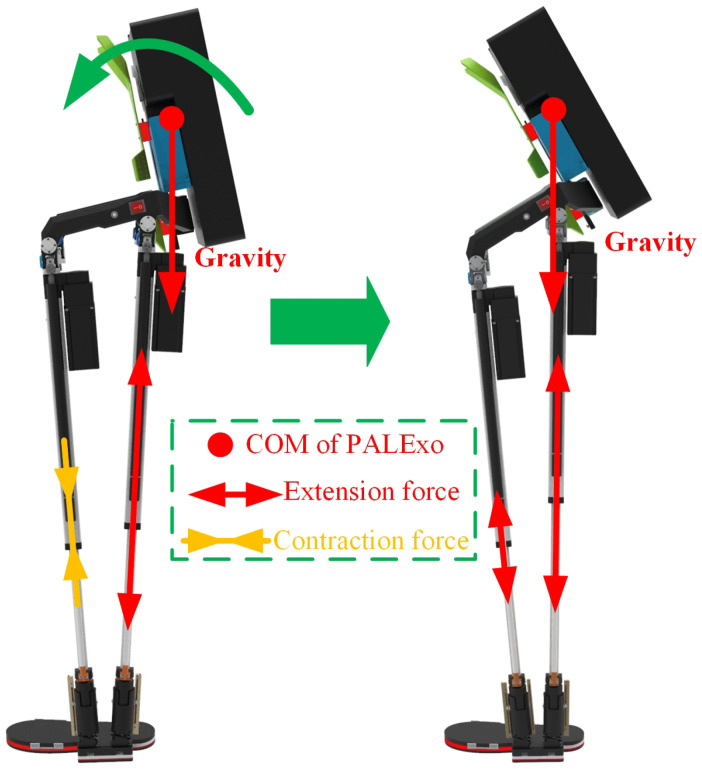
Process of adjusting COM at the initial moment when PALExo is energized.

The main parameters of the three selected springs are shown in Table 3. It should be especially explained that the selected CFSs of the SEMS in the two chains of PALExo’s single leg are different, because of the different output contraction force requirements of the two actuators in the two chains. For example, PALExo is usually hung on a fixed frame when it is not working. The loads on the back of PALExo leave the COM of PALExo located behind the two actuators, as shown in Figure 6. The front actuator is supposed to output a relatively large contraction force to adjust PALExo’s COM at the initial moment when the exoskeleton is energized.

### 3.3. Stiffness Identification

Due to installation errors and nominal parameter errors, it is almost impossible that the theoretical stiffness and actual stiffness of the gas spring and SEM are exactly the same. Therefore, stiffness identification of the gas spring and SEM are necessary. The stiffness identification of the SEM has been carried out in [37].

The telescopic cylinder is applied to identify the stiffness of the gas spring. Firstly, the telescopic cylinder is controlled to extend and contact at a constant speed of 0.01 m/s. The current of the motor is measured to calculate the output force of the telescopic cylinder, which is equal to the friction of the telescopic cylinder. Secondly, the gas spring is installed in parallel with the telescopic cylinder to constitute a PEA. The PEA is controlled to extend and contact at a constant speed of 0.01 m/s, and the current of the motor is measured. Finally, the extension force of the gas spring at different positions can be calculated. At a certain position, the output force of the telescopic cylinder is equal to the vector sum of the friction of the telescopic cylinder, the extension force of the gas spring and the friction of the gas spring, as shown in Equations (6) and (7).

(6) $$F_{t}^{e}=F_{g}-f_{t}^{e}-f_{g}^{e},$$

(7) $$F_{t}^{c}=F_{g}+f_{t}^{c}+f_{g}^{c},$$

where, $F_{t}^{e}$ and $F_{t}^{c}$ represent the output force of the telescopic cylinder during its extension motion and contraction motion, respectively. $F_{g}$ represents the extension force of the gas spring, $f_{t}^{e}$ and $f_{t}^{c}$ represent the friction of the telescopic cylinder during extension motions and contraction motions, respectively. $f_{g}^{e}$ and $f_{g}^{c}$ represent the friction of the gas spring during the extension motion and contraction motion, respectively.

We assume that the friction of the gas spring is unrelated to the motion directions, i.e., $f_{g}^{e}=f_{g}^{c}$. Combining Equations (6) and (7), the extension force of the gas spring can be obtained, as shown by Equation (8).

(8) $$F_{g}=\left(F_{t}^{e}+F_{t}^{c}+f_{t}^{e}+f_{t}^{c}\right)/2,$$

**Figure 7:**
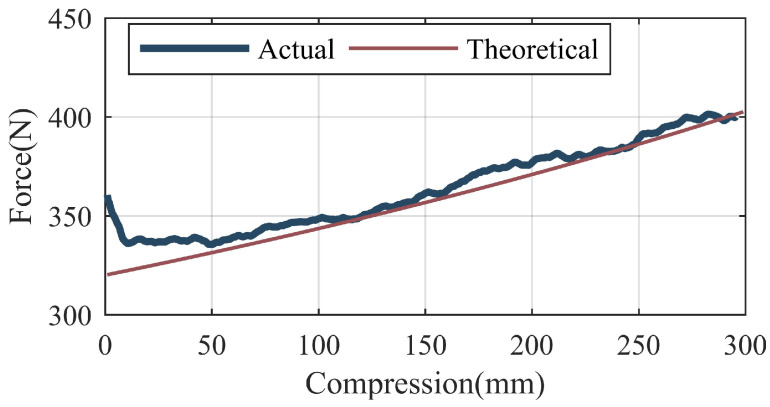
Result of the stiffness identification of the gas spring.

The result of the stiffness identification of the gas spring are shown in Figure 7.

## 4. Control of the SPEA

The SPEA introduced above is regarded as a force source during the movement of PALExo. The target force of the SPEA is obtained from a higher-level control strategy of PALExo. With a perfect force source, impedance is zero (completely backdriveable), stiction is zero, and bandwidth is infinite [39]. For human–robot interactions, the controller of the SPEA should meet the following performance objectives. (1) It should reduce the mechanical impedance of the SPEA by compensating for inertia and the friction of the actuator. (2) It should make the SPEA precisely generate force following the target force curve. (3) It should guarantee the robust performance of the SPEA while interacting with a human [11]. At present, reports on SPEAs and PEAs mostly focus on structural innovation, and there are few studies on the force control of SPEAs and PEAs. In this paper, the controller of the SPEA is designed with reference to the force control methods of SEAs, which have been widely researched.

Pratt G A et al. introduced the concept of SEAs and presented a passive control concept that is based on several feed-forward compensation terms and a PID controller [6]. In this controller, the motor is treated as a torque source. Wyeth G et al. presented a cascaded controller with an inner velocity loop, treating the motor as a velocity source [9]. The cascaded controllers with an inner velocity loop and an inner position loop have both been widely investigated in the past twenty years [11,40,41]. To guarantee the stability and robustness of the coupled human–robot system, Andrea C et al. proposed a force control approach based on SMC [42].

The environment’s dynamics, which are usually uncertain and time variant, have an impact on the force control performance. Several solutions have been proposed to mitigate the impact of the human or the environment, three classes of which are explained. (1) DOBs have been widely used in many SEA force controllers, considering unknown environment dynamics as disturbances to be rejected [5,11,43,44]. (2) The human and environment are considered in the dynamic model in some model-based force controllers [19,45,46]. (3) Positive acceleration feedback from the robot joint is used to mitigate the impact of the human or environment. The feedback gain of this solution is difficult to select. To avoid overestimation in this solution, the multiplicative gain of acceleration feedback has to be sufficiently small and other acceleration feedback terms cannot be included in the controller, such as most DOBs [47]. The latter two solutions rely more on prior knowledge of environmental parameters, such as the inertia of the load. However, the working conditions of the PALExo SPEA are complex and change quickly. Additionally, there are many disturbances when the exoskeleton is working. In this paper, a DOB is selected to reduce the adverse effects of environmental changes and disturbances to force control.

**Figure 8:**
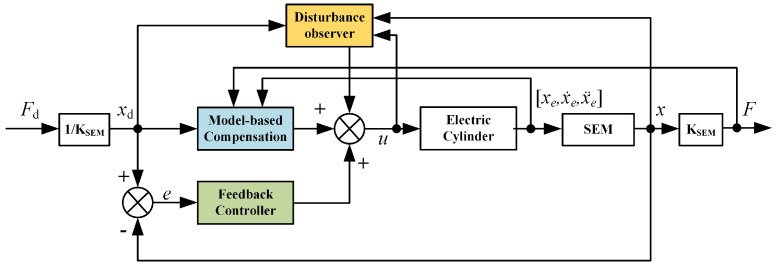
Block diagram of the SPEA control system.

In conclusion, a SPEA force control method is designed as shown in Figure 8. The force control system is composed of model-based compensation, a feedback controller, and a disturbance observer, which will be introduced in the rest of this section.

### 4.1. Model-Based Compensation

The dynamic model of each electric cylinder can be formulated as follows:

(9) $$u=M\cdot {\ddot{x}}_{e}+B\left(x_{e},{\dot{x}}_{e}\right)\cdot {\dot{x}}_{e}+f_{t}\left(x_{e},{\dot{x}}_{e}\right)+G+d\left(t\right),$$

where, *u* is the input torque from the motor, $x_{e}$, ${\dot{x}}_{e}$ and ${\ddot{x}}_{e}$ are the displacement, velocity, and acceleration of the electric cylinder, respectively. *M* is the mass of the electric cylinder, $B(x_{e},{\dot{x}}_{e})$ is the Coriolis and centrifugal force coefficient, $f_{t}\left(x_{e},{\dot{x}}_{e}\right)$ is the friction force, *G* is the gravitational force, d(t) is the disturbance from the environment.

In Equation (9), all forces from other elements are included in d(t). However, some forces in d(t) acting on the electric cylinder are known, including forces from the SEM, which are forces from the gas spring. The forces from the gas spring are composed of an extension force identified in Section 3 and the friction force of the gas spring. Thus the dynamic model of each electric cylinder can be modified, represented as Equations (10) and (11), where the friction forces of the gas spring and electric cylinder are combined.

(10) $$u=M\left(x_{e}\right)\cdot {\ddot{x}}_{e}+B\left(x_{e},{\dot{x}}_{e}\right)\cdot {\dot{x}}_{e}+G+F_{s}+F_{g}+f+d\left(t\right),$$

(11) $$f=f_{g}+f_{t},$$

where, $F_{s}$ is the force from the SEM, $F_{g}$ is the extension force of the gas spring, *f* is the friction force of both the telescopic cylinder and the gas spring, and $f_{g}$ is the friction force of the gas spring.

Friction is a complex phenomenon that depends on many physical parameters and working conditions, for which none of the available models can claim general validity [48]. Taking into account the capability of replicating stiction, the Stribeck effect, and pre-sliding displacement, the LuGre friction model [49] is selected to identify the friction force of the electric cylinder and the gas spring. The LuGre friction model is based on the bristle model, which supposes there are many bristles at the contact interface. The friction force varies prior to slip and is caused by the elastic deformation of these bristles. Once the bristles reach their maximum deformation, slip starts to occur [50]. The friction force of the LuGre model is defined as follows:

(12) $$f=\sigma_{0}z+\sigma_{1}\dot{z}+\sigma_{2}{\dot{x}}_{e},$$

where *z* is the state variable that represents the state of deformation of the bristles, $\sigma_{0}$ is the stiffness of the bristles, $\sigma_{1}$ is the viscous damping of the bristles, and $\sigma_{2}$ is the viscous damping of the contact. $\dot{z}$ is the time derivative of z and it is expressed as Equations (13) and (14).

(13) $$\dot{z}={\dot{x}}_{e}\left(1-\frac{\sigma_{0}z}{g(v)}sgn\left({\dot{x}}_{e}\right)\right),$$

(14) $$g\left(v\right)=F_{d}+\left(F_{s}-F_{d}\right)e^{-{\left({\dot{x}}_{e}/v_{s}\right)}^{2}},$$

where $F_{d}$ is the Coulomb friction force, $F_{s}$ is the stiction force, and $v_{s}$ is the Stribeck velocity.

To simplify the model, the relative motion between the sliding surfaces can be considered to be quasi-static, namely $\dot{z}=0$. Modifying the LuGre model gives Equation (15).

(15) $$f=\left(F_{d}+\left(F_{s}-F_{d}\right)e^{-{\left({\dot{x}}_{e}/v_{s}\right)}^{2}}\right)sgn\left({\dot{x}}_{e}\right)+\sigma_{2}{\dot{x}}_{e},$$

The friction force can be obtained by identifying the parameters, including $F_{d}$, $F_{s}$, $v_{s}$, and $\sigma_{2}$.

**Figure 9:**
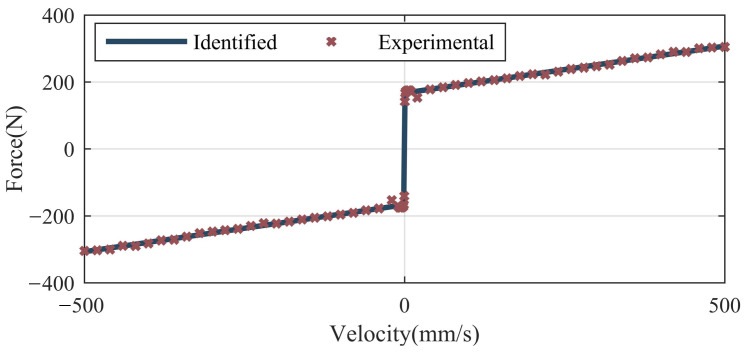
Friction force curve of the electric cylinder and the gas spring.

To identify the friction force, the electric cylinder is controlled to move at different constant speeds and the motor torque is recorded. The friction force can be obtained by subtracting the extension force of the gas spring from the motor torque. A method based on a genetic algorithm has been introduced in [37] to identify the friction force of the electric cylinder. The same method is used to identify the total friction force of both the electric cylinder and gas spring in this paper. For the sake of simplicity, we will not repeat it. The friction force curve identified is shown in Figure 9. The identified LuGre model is expressed as Equation (16).

(16) $$f=\left(166.99+\left(58.29-166.99\right)e^{-{\left({\dot{x}}_{e}/0.42\right)}^{2}}\right)sgn\left({\dot{x}}_{e}\right)+0.28{\dot{x}}_{e}$$

### 4.2. Force-Feedback Control

A cascaded PID controller with an inner velocity loop and a sliding mode controller are widely used in the feedback control of SEAs. In this paper, these two controllers are introduced and their effects compared experimentally.

(1) Cascaded PID controller with an inner velocity loop

A cascaded PID controller is designed, which is composed of an inner velocity loop as shown in Equation (17) and an outer position loop as shown in Equation (19).

(17) $$u_{fb}=K_{vp}e_{v}+K_{vd}{\dot{e}}_{v}+K_{vi}\int e_{v},$$

(18) $$e_{v}=v_{d}-v,$$

(19) $$v_{d}=K_{xp}e+K_{xd}\dot{e},$$

where, $u_{fb}$ is the control input from the feedback controller. $K_{vp}$, $K_{vd}$, and $K_{vi}$ are the proportional gain, derivative gain, and integral gain in the inner velocity loop, respectively. $e_{v}$ and *e* are the velocity error and position error, respectively. $v_{d}$ and *v* are the desired velocity and actual velocity in the inner velocity loop, respectively. $K_{xp}$ and $K_{xd}$ are th proportional gain and derivative gain in the outer position loop, respectively.

Combining the cascaded PID controller and model-based compensation in Equation (10), the force controller of the SPEA is obtained as shown in Equation (20).

(20) $$u=M\cdot {\ddot{x}}_{e}+B\cdot {\dot{x}}_{e}+G+F_{s}+F_{g}+f+d+u_{fb}$$

(2) Sliding mode controller

A similar sliding mode controller to that in [37] is designed. The sliding mode surface is designed as:

(21) $$s=\dot{e}+ce,$$

where *c* is a positive constant. Combining this with Equation (10), the approach law is obtained:

(22) $$\dot{s}=c\dot{e}+{\ddot{x}}_{d}-\left(u-B\cdot {\dot{x}}_{e}-G-F_{s}-F_{g}-f-d\right)/M,$$

To improve the dynamic quality of approaching motion, the approach law is designed as:

(23) $$\dot{s}=-\eta \cdot sgn\left(s\right)-ks,$$

where η and *k* are two positive constants. Combining this with Equations (22) and (23), the force controller based on sliding mode control of the SPEA is obtained, as shown in Equation (24).

(24) $$u=M\left(c\dot{e}+{\ddot{x}}_{d}+\eta \cdot sgn\left(s\right)+ks\right)+B\cdot {\dot{x}}_{e}+G+F_{s}+F_{g}+f+d,$$

To alleviate the jitter problem of the sliding mode controller, Equation (24) is modified by replacing the symbolic function sgn(s) with s/(|s|+δ):

(25) $$u=M(c\dot{e}+{\ddot{x}}_{d}+\eta \cdot s/(|s|+\delta )+ks)+B\cdot {\dot{x}}_{e}+G+F_{s}+F_{g}+f+d,$$

where δ is a small positive constant.

### 4.3. Disturbance Observer

It has been verified that disturbance observers are conducive to SEA force control [5,11]. According to Equation (10), the disturbance can be easily expressed as:

(26) $$d=u-M\cdot {\ddot{x}}_{e}-B\cdot {\dot{x}}_{e}-G-F_{s}-F_{g}-f$$

A simple DOB can be designed as:

(27) $$\dot{\hat{d}}=-L\left(x_{e},{\dot{x}}_{e}\right)\hat{d}+L\left(x_{e},{\dot{x}}_{e}\right)\left(u-M\cdot {\ddot{x}}_{e}-B\cdot {\dot{x}}_{e}-G-F_{s}-F_{g}-f\right),$$

where $\hat{d}$ is the estimated value of the disturbance and $L(x_{e},{\dot{x}}_{e})$ is the observer gain. However, accurate acceleration is difficult to obtain; there is significant noise using a second derivative of the position measurements. To solve this problem, a nonlinear observer [51] is used in this paper.

An auxiliary variable vector is defined:

(28) $$z=\hat{d}+p\left(x_{e},{\dot{x}}_{e}\right),$$

(29) $$p\left(x_{e},{\dot{x}}_{e}\right)=a{\dot{x}}_{e},$$

where *a* is a positive constant. The function $L(x_{e},{\dot{x}}_{e})$ is determined by following equation:

(30) $$L\left(x_{e},{\dot{x}}_{e}\right)M{\ddot{x}}_{e}=\frac{dp(x_{e},{\dot{x}}_{e})}{dt},$$

Thus,

(31) L=a/M,

Combining Equations (27), (28) and (30),

(32) $$\begin{array}{cc} \; & \dot{z}=\dot{\hat{d}}+\frac{dp(x_{e},{\dot{x}}_{e})}{dt}=-L\hat{d}+L\left(u-M\cdot {\ddot{x}}_{e}-B\cdot {\dot{x}}_{e}-G-F_{s}-F_{g}-f\right)+LM{\ddot{x}}_{e}\\ \; & =-L\left(z-p\left(x_{e},{\dot{x}}_{e}\right)\right)+L\left(u-B\cdot {\dot{x}}_{e}-G-F_{s}-F_{g}-f\right) \end{array},$$

The DOB is designed as:

(33) $$\dot{z}=-az/M+a\left(u-B\cdot {\dot{x}}_{e}-G-F_{s}-F_{g}-f+a{\dot{x}}_{e}\right)/M,$$

(34) $$\hat{d}=z-a{\dot{x}}_{e},$$

In considering the parameter errors, Equation (33) is modified as:

(35) $$\dot{z}=-az/M_{0}+a\left(u-B_{0}\cdot {\dot{x}}_{e}-G_{0}-F_{s0}-F_{g0}-f_{0}+a{\dot{x}}_{e}\right)/M_{0},$$

where, $M_{0}$, $B_{0}$, $G_{0}$, $F_{s0}$, $F_{g0}$, and $f_{0}$ are estimates of *M*, *B*, *G*, $F_{s}$, $F_{g}$, and *f*, respectively.

We define the observer error as:

(36) $$e_{d}=d-\hat{d},$$

Assuming the disturbance is a slow time-varying signal and $\dot{d}=0$, combining Equations (26), (35) and (36) gives:

(37) $$\begin{array}{cc} \; & {\dot{e}}_{d}=-\dot{\hat{d}}=-\dot{z}+a{\ddot{x}}_{e}\\ \; & =\frac{a}{M}z-\frac{a}{M}\left(u-B_{0}{\dot{x}}_{e}-G_{0}-F_{s0}-F_{g0}-f_{0}+a{\dot{x}}_{e}\right)+a{\ddot{x}}_{e}\\ \; & =\frac{a}{M}z-\frac{a}{M}\left(M{\ddot{x}}_{e}+B{\dot{x}}_{e}+G+F_{s}+F_{g}+f+d-B_{0}{\dot{x}}_{e}-G_{0}-F_{s0}-F_{g0}-f_{0}+a{\dot{x}}_{e}\right)+a{\ddot{x}}_{e} \end{array},$$

Defining that $\Delta H=\left(M-M_{0}\right){\ddot{x}}_{e}+\left(B-B_{0}\right){\dot{x}}_{e}+G-G_{0}+F_{s}-F_{s0}+F_{g}-F_{g0}+f-f_{0}$, Equation (37) can be modified as:

(38) $${\dot{e}}_{d}=\frac{a}{M}\left(\hat{d}-d-\Delta H\right)=-\frac{a}{M}e_{d}-\frac{a}{M}\Delta H,$$

Solving Equation (38), $e_{d}$ can be obtained as shown in Equation (39).

(39) $$e_{d}=Ce^{-(a/M_{0})t}-\Delta H,$$

where, *C* is a constant. It can be shown that the observer is globally asymptotic because a/M is positive. The exponential convergence rate can be specified by choosing *a*:

(40) $$\lim_{t\to \infty }\;e_{d}=-\Delta H,$$

(41) $$\lim_{t\to \infty }\;\hat{d}=d+\Delta H,$$

Equation (41) represents that the DOB can not only estimate the external disturbance but also compensate for the parameter errors.

## 5. Experiments

**Figure 10:**
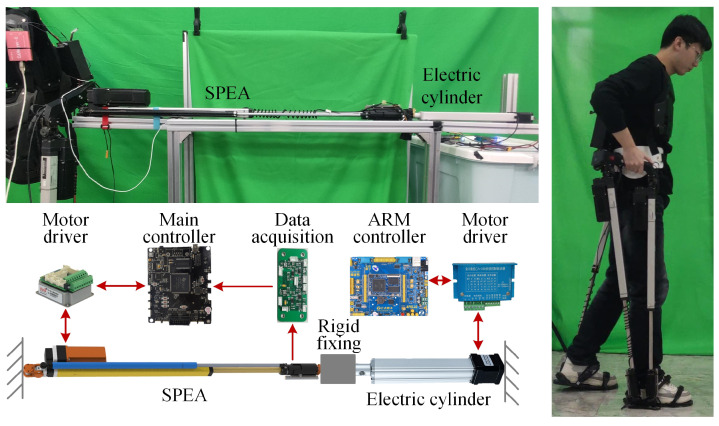
Experimental conditions.

To verify the effects of the above two SPEA control strategies, several experiments are carried out under the experimental conditions shown in Figure 10. An electric cylinder (IP42X-300: SKISIA Inc., Wuxi, China) is used for testing the performance of the control strategies. The electric cylinder and the SPEA are all fixed on the same aluminum frame at their tails and are fixed together at their heads. The SPEA is controlled by the main controller of the PALExo and a motor driver (SimplIQ SOL-WHI 20/60E: Elmo Inc., Petach-Tikva, Israel). The AOC of the SEM is collected by a data acquisition circuit board. The electric cylinder is controlled by an ARM-based controller (Apollo STM32F767 development board: Alientek Inc., Guangzhou, China) and a motor driver for the stepping motor (CA-230: YASKAWA Inc., Kitakyushu, Japan).

**Figure 11:**
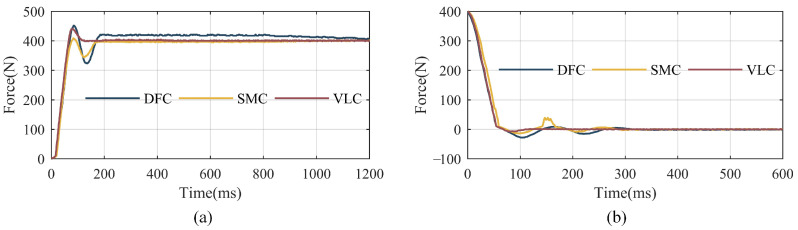
The response curves of step signal with different control strategies. (**a**) Response curves when the target force is 400 N; (**b**) response curves when the target force is 0 N. DFC, SMC, and VLC respectively indicate the direct force-control based on the PID with a DOB, sliding mode control with the DOB mentioned in Section 4, and a cascaded PID control with an inner velocity loop and the DOB mentioned in Section 4.

Several experiments are carried out to test the response speed of the SPEA with different control strategies. The electric cylinder is kept stationary and the target output force is set as 400 N. The output force of the SEM is collected from the SPEA, receiving control commands at the end of its movements, as shown in Figure 11a. Three control strategies are applied in the experiments for comparison, which are direct force-control based on the PID with a DOB (expressed as DFC in Figure 11), sliding mode control with the DOB mentioned in Section 4 (expressed as the SMC in Figure 11), and a cascaded PID control with an inner velocity loop and the DOB mentioned in Section 4 (expressed as the VLC in Figure 11). The time is calculated by sampling frequency and data number.

The stiffness of the SEM is variable when the AOC is changing and the control strategies of the SPEA are supposed to apply to different stiffnesses. To verify the adaptability of the control strategies, a step signal from 400 N to 0 N is set as the target forces of the SPEA and the response curves are recorded and shown in Figure 11b.

It can be seen from the response curves shown in Figure 11a that the SPEA can always achieve accurate target force with the three different control strategies. However, it needs about 1 s to eliminate the static error of the direct force-control based on a PID with a DOB, which is probably caused by an integral term in the PID controller. The curve of the cascaded PID control with an inner velocity loop and a DOB stabilize around the target value firstly and the time it costs is about 120 ms.

Figure 11b shows that the response curves of DFC and SMC jitter more obviously than that of Figure 11a with same parameters. However, the curve of VLC can also reach the target force quickly and stably. It indicates that VLC has better adaptability to different SEM stiffnesses than DFC or SMC.

**Figure 12:**
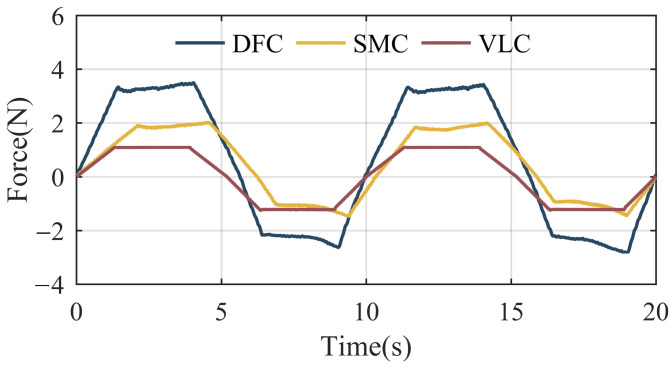
Interaction force curves with different control strategies.

The SPEA in the swinging legs of PALExo is expected to follow the movement of a human swinging their feet, with as little interaction force as possible. To verify the following performance of the SPEA, the electric cylinder is controlled to do reciprocating motion at a speed of 60 mm/s. The interaction force between the SPEA and the electric cylinder is calculated by the AOC of the SEM with three different control strategies, as shown in Figure 12.

It can be seen from Figure 12 that the maximum values of the interaction force with DFC, SMC, and VLC are 3.2 N, 2 N, and 1.1 N, respectively. The cascaded PID control with an inner velocity loop and a DOB performs best in above experiments among the three different control strategies.

**Figure 13:**
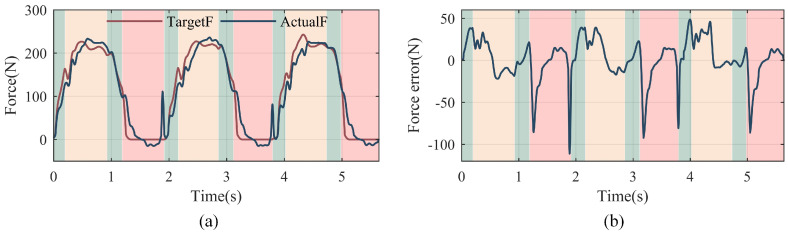
Output force curves of the SPEA when the exoskeleton is walking. (**a**) Target force and actual force curves. (**b**) The force tracking error curve. The red curve indicates the target force based on dynamic calculation and the blue curve indicates the actual output force of the SPEA. The green background indicates the walking state with both legs standing. The yellow background indicates the walking state with the left leg standing and the right leg swinging. The red background indicates the walking state with the left leg swinging and the right leg standing.

To verify the effect of VLC when used in the exoskeleton, the output force of the SPEA of PALExo is recorded when a wearer is walking with PALExo, as shown in Figure 13. The SPEA at the left rear of PALExo is taken as the experimental subject. The target force of the SPEA is obtained by dynamic calculation.

The target force and actual force curves in Figure 13 show that the SPEA can track the target force when the exoskeleton is walking. The tracking error is caused by not only the performance of VLC but also sudden disturbances, especially when the left foot hits and leaves the ground. It shows that the ability to resist high-frequency disturbances of the control strategy needs to be improved.

## 6. Conclusions

In this paper, a SPEA is proposed for an exoskeleton named PALExo. Due to special working conditions, the actuators in PALExo need to provide large force in the direction of elongation and in the standing phase, but they need only provide little force in the direction of shortening in the swinging phase. A gas spring is installed in parallel with an electric cylinder to improve its supporting capacity and a variable-stiffness SEM is installed in series with the electric cylinder to improve its compliance. A control strategy based on a cascaded PID control strategy with an inner velocity loop and a DOB is proposed to control the output force of the SPEA. By several experiments, the effect of the force control strategy is verified with respect to its stepping response and its following the movement of human. In addition, the force control strategy of SPEA has good adaptability to different SEM stiffnesses.

Although some achievements have been made in this paper, the research has some limitations. It is difficult for the SPEA control strategy to resist high-frequency disturbances. Additionally, the compliance of the SPEA’s output cannot be controlled, as it depends on the stiffnesses of the elastic elements. Our future work will focus on improving the suppression effect of high-frequency disturbances and controlling the compliance of the SPEA’s output with impedance control, according to the requirements of PALExo.

## Figures and Tables

**Table 1:** Main parameters of the PC25 telescopic cylinder and APM-SB04A motor.

**Telescopic Cylinder**	**Maximum Force**	**Stroke**	**Maximum Speed**	**Lead**	**Section Size**
	1250 N	0.4 m	1.33 m/s	0.01 m	0.034 m × 0.034 m
**Motor**	**Nominal Power**	**Nominal Torque**	**Nominal Speed**	**Nominal Voltage**	**Nominal Current**
	400 W	1.27 N·m	60 r/s	48 V	9.37 A

**Table 2:** This is a table caption. Tables should be placed in the main text near to the first time they are cited.

$F_{0}$	*L*	$S_{\mathrm{pc}}$	$S_{\mathrm{rod}}$
320 N	0.45 m	$2.54\times 10^{-4}\;m^{2}$	$7.85\times 10^{-5}\;m^{2}$

**Table 3:** Main parameters of the selected springs in the SEM.

Location of SEM	Springs	Outer Diameter	Wire Diameter	Initial Length	Stiffness Coefficient
Front	Stiff EFS	28.2 mm	3.8 mm	80 mm	31.03 N/mm
	Soft EFS	16.5 mm	1.5 mm	110 mm	0.83 N/mm
	CFS	14.5 mm	2.0 mm	22 mm	20.48 N/mm
Back	Stiff EFS	28.2 mm	3.8 mm	80 mm	31.03 N/mm
	Soft EFS	16.5 mm	1.5 mm	110 mm	0.83 N/mm
	CFS	9.9 mm	0.8 mm	22 mm	1.73 N/mm

## Abbreviations

The following abbreviations are used in this manuscript:

SEA	series elastic actuators
DOB	disturbance observer
PD	proportional derivative
VSA	variable stiffness actuator
NLS	nonlinear spring
PEA	parallel elastic actuator
SPEA	series–parallel elastic actuator
SMC	sliding mode control
PID	proportional–integral–derivative
DOF	degree of freedom
COM	center of mass
CE	contractile element
SE	series elastic element
PE	parallel elastic element
AOC	amount of compression
SEM	series elastic module
EFS	extension-force spring
CFS	contraction-force spring
